# Sustainable Biocatalytic Procedure for Obtaining New Branched Acid Esters

**DOI:** 10.3390/ma14226847

**Published:** 2021-11-13

**Authors:** María Claudia Montiel, Miguel Asensi, Silvia Gimeno-Martos, Fuensanta Máximo, Josefa Bastida

**Affiliations:** Chemical Engineering Department, Faculty of Chemistry, Campus Mare Nostrum, University of Murcia, 30100 Murcia, Spain; cmontiel@um.es (M.C.M.); miguel.asensil@um.es (M.A.); silvia.gimeno@um.es (S.G.-M.); fmaximo@um.es (F.M.)

**Keywords:** biocatalysis, branched-chain acid ester, solvent-free, process productivity, green metrics, economic assessment

## Abstract

Biocatalytic synthesis of 2-ethylhexyl 2-methylhexanoate is described in this work for the first time. This branched-chain ester is suitable for use at low temperatures in numerous applications. The immobilized lipase Novozym^®^ 435 has demonstrated its ability to catalyze the ester synthesis from 2-ethylhexanol and 2-methylhexanoic acid in a solvent-free medium. The high reaction times that are required result in a loss of alcohol by evaporation, which must be compensated for with an excess of this substrate if high conversions are to be achieved. Therefore, two strategies are established: 70 °C with a 10% excess of alcohol, which requires a longer operating time and provides conversions of 97%, and 80 °C with a 20% excess of alcohol, which allows for the achievement of a 99% conversion in a shorter time. The optimal reaction conditions have been chosen based on reusability of the enzyme, process productivity, green metrics and preliminary economic study. When the synthesis is carried out under the best conditions (70 °C, 10% molar excess of alcohol and six uses of the immobilized enzyme) a productivity of 203.84 kg product × kg biocatalyst^−1^ is attained. The biocatalytic procedure matches many of the objectives of “green chemistry” and is suitable to be scaled up and used in industrial manufacturing.

## 1. Introduction

Esters are widely used compounds with a great diversity of compositions, structures and properties. They have many different applications as additives for plastics, resins, explosives, biodiesel, lubricants, paper and food, as well as in the cosmetic industry.

In the case of long-chain esters from saturated fatty acids, they have the facility to crystallize at low temperatures. Consequently, these esters show high melting and boiling points and are fluid in a small temperature range. The presence of unsaturated fatty acids increases the fluidity of esters, but it may reduce their chemical stability and, therefore, they are easily oxidized under normal conditions. In this way, branched-chain esters (BCEs) show better properties at low temperatures, since the branches contribute to reduced pour points and prevent crystallization [1,2]. BCEs can be used as an environmentally friendly alternative due to their high biodegradability, and moreover, they can be obtained from natural raw materials by biocatalytic processes without a high level of energy consumption [3].

In the development of these processes, lipases are used as biocatalysts because they can catalyze the synthesis of a wide variety of esters from different substrates operating under mild conditions. Among the numerous advantages of lipases, the ability to be activated both in water-free and in solvent-free mediums, resulting in products with high purity without downstream processing steps, stands out. Indeed, lipase immobilization increases operational stability as well as facilitates its separation and reuse. Therefore, lower-cost processes according to “green chemistry principles” can be developed, which are more efficient and environmentally friendly than chemical ones [1].

Many studies have described the biocatalytic synthesis of BCEs using lipases from different sources and with different substrates [4,5,6,7]. In these mentioned articles, authors have worked with linear acids and branched alcohols as substrates and with different immobilized commercial *Candida antarctica* lipase B (CalB) as enzymes.

However, the enzymatic synthesis becomes more complex when the branched substrate is the acid, due to limitations close to the active site of the enzyme. Branched acid esters are important raw materials for the cosmetic industry, where they are used as emollient and emulsifier additives [8,9]. In particular, esters of 2-ethylhexanoic acid are widely used ingredients in the cosmetic and pharmaceutical industries since they improve the spreading and wetting behavior of the product that contains them, especially when applied to human skin [10]. In the bibliography, there are only three previous works where the biocatalytic production of 2-ethylhexanoic acid esters with different alcohols [10,11] and the use of supercritical CO_2_ as a solvent medium [12] is described. The low efficiency of these enzymatic processes is evidenced by the high reaction times [10,11], the high concentration of biocatalyst required [10], the low concentrations of substrates [10], the use of solvents [10,12] and the low conversions achieved [10,12].

Other researchers have used CalB variants to obtain esters from different branched carboxylic acids as 3,5,5-trimethylhexanoic acid and 2-ethylhexanoic acid [13]. None of the lipase variants tested have been able to improve the activity of wild-type CalB towards 2-ethylhexanoic acid. On the other hand, when 3,5,5-trimethylhexanoic acid was used as substrate, excellent results were obtained with the majority of lipases tested. These results evidence that the length and position of the branched-chain of the carboxylic acid are critical for the expression of the catalytic activity of CalB. Therefore, the development of a successful biocatalytic synthesis of branched acid esters requires the study of branched acids other than 2-ethylhexanoic acid.

**Figure 1:**
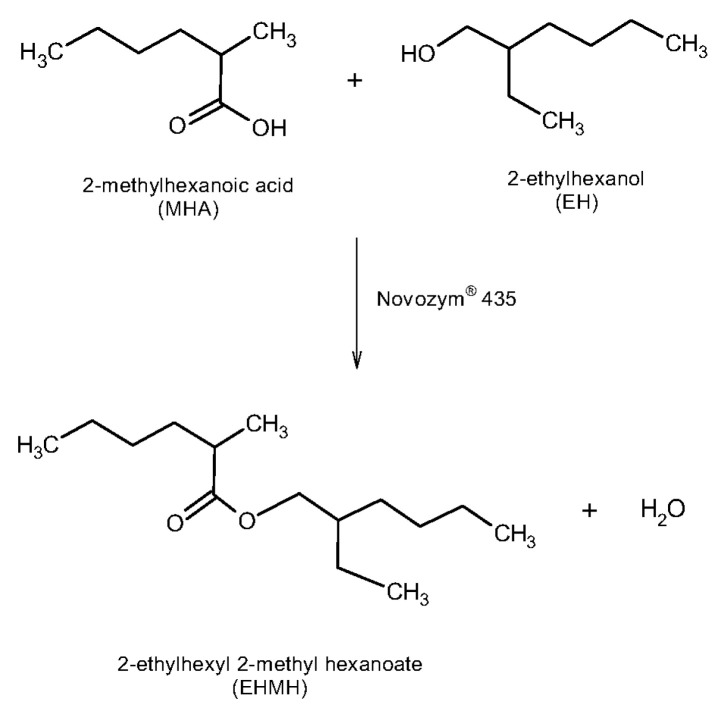
Reaction scheme of the biocatalytic synthesis of 2-ethylhexyl 2-methylhexanoate.

Thus, the aim of this work is to study the synthesis of a branched-chain ester (2-ethylhexyl 2-methylhexanoate, EHMH) obtained from two branched substrates: 2-methylhexanoic acid (MHA) and 2-ethylhexanol (EH). The well-known commercial immobilized lipase, Novozym^®^ 435, was used as biocatalyst and the reactions were carried out in a solvent-free medium. The reaction scheme is shown in Figure 1. In order to analyze the benefits of this enzymatic process and to evaluate the viability of an industrial implementation to produce it as a cosmetic additive, some indicators regarding the enzyme reusability, productivity, sustainability and economy of the process have been calculated.

## 2. Materials and Methods

### 2.1. Materials

The biocatalyst used was Novozym^®^ 435, a commercial *Candida antarctica* lipase B (CalB), immobilized on a macroporous acrylic resin Lewatit VP OC 1600, which was kindly donated by Novozymes Spain S.A. Substrates 2-methylhexanoic acid (MHA, 99%) and 2-ethylhexanol (EH, 99.6%) were supplied by Sigma-Aldrich Co. (St. Louis, MO, USA) All other reagents and products were analytical-grade.

### 2.2. Enzymatic Synthesis

The reaction synthesis was carried out in an open-air glass-jacketed tank reactor (Vidra FOC 505/2, 50 mL) provided with a vertical paddle stirrer (Ika Nanostar 7.5 digital) at 350 rpm. When the operating temperature described below was reached, substrates were introduced in the reactor and the enzymatic synthesis started when the biocatalyst was added in a concentration of 2.5% (*w/w*, referring to substrates). Samples were taken at different intervals (stirring was stopped a few seconds befoProcesse this), to follow the reaction course by measuring the acid value and dissolved in absolute ethanol for gas chromatography (GC) analysis. Several experiments were carried out under the reaction conditions described in Table 1.

### 2.3. Acid Value Determination

The acid value (AV) represents the number of milligrams of potassium hydroxide necessary to neutralize free acids contained in 1 g of sample [14]. An automatic titrator, Metrohm 888 Titrando, was used.

The conversion based on acid value was calculated as:

$$\mathrm{Conversion}\;\left(\%\right)=\frac{{\left(\mathrm{AV}\right)}_{i}-{\left(\mathrm{AV}\right)}_{t}}{{\left(\mathrm{AV}\right)}_{i}}\times 100$$

where (AV)_i_ is the acid value at the beginning of the reaction and (AV)_t_ is acid value of the product at time = t. The results presented in this work are the mean of three different measurements and errors were always lower than 10%.

### 2.4. Gas Chromatography Analysis

Substrates and product concentrations were determined by injecting 1 μL of diluted sample in a 7820A gas chromatographer (GC) from Agilent, equipped with a flame ionization detector (FID) and a silica capillary column (HP-5 Agilent Technologies; 30 m × 0.32 mm × 0.25 μm). The analysis conditions were: the injector temperature was 250 °C, with a split ratio of 2:1, and the carrier gas used was nitrogen, at a flow rate of 1 mL × min^−1^. The oven temperature was maintained at 80 °C for one minute, then increased to 120 °C with a rate of 75 °C × min^−1^ and then held for one minute. Then, the oven temperature was increased again to 290 °C at a ramping rate of 20 °C × min^−1^, a temperature which was held for 3.5 min. The detector temperature was 300 °C. The samples’ composition was determined by using methyl myristate as the internal standard.

### 2.5. Recovery of Immobilized Lipase

The reuse of the biocatalyst was studied by recycling the immobilized lipase several times. For this purpose, the bulk of the product was removed after the biocatalyst was settled down. Additionally, the biocatalyst was recovered by rinsing three times with acetone, filtering and air-drying for 24 h at room temperature before using them without further purification in another batch with new fresh reagents.

## 3. Results and Discussion

### 3.1. Biocatalytic Synthesis of 2-Ethylhexyl 2-Methylhexanoate

The enzymatic synthesis of EHMH has still not been described in the bibliography and, therefore, the authors first tried to obtain it using experimental conditions based on the experiences acquired from previous studies [4,5,6,7]. For this purpose, the discontinuous tank reactor was provided with vertical agitation to avoid damage to the biocatalyst that was used, Novozym^®^ 435 [15]. The reaction conditions were: solvent-free medium, 70 and 80 °C, 350 rpm, a substrate molar ratio of 1:1 and 2.5% (*w/w*) of biocatalyst.

**Figure 2:**
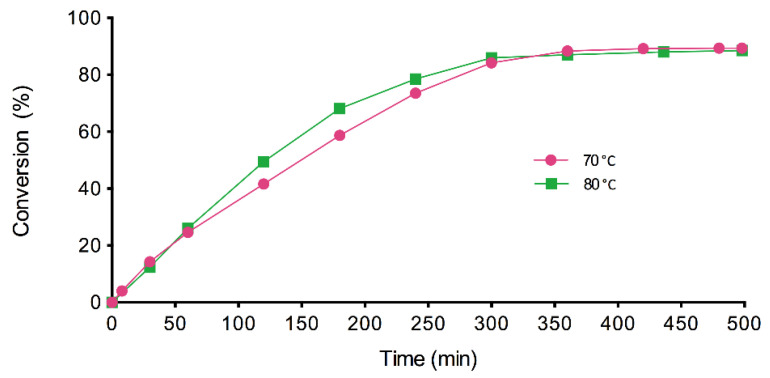
Conversion values of EHMH with time at 70 °C and 80 °C, 350 rpm, 2.5% (*w/w*) of biocatalyst and a substrate molar ratio of 1:1.

The results obtained in these preliminary experiments are shown in Figure 2, where the evolution of the conversion (%) with time is plotted. These results reveal that the solvent-free biocatalytic synthesis of EHMH has been successfully conducted for the first time in the literature. Besides, they allow for the expansion of a field of study to the development of sustainable processes for the synthesis of new BCEs. As it can be noticed in the figure, the reaction rates at 1 h were similar for both temperatures. After that, the enzymatic reaction rate was higher at 80 °C. However, after 7 h, the conversion was slightly higher at 70 °C than at 80 °C (89.38% and 88.57%, respectively). The conversions achieved are not high enough to avoid the costly purification operations necessary to obtain a suitable product for cosmetic uses [16]. On the other hand, Figure 2 also shows that the enzymatic reaction stops after 6 h of reaction, which could be attributed to an enzyme deactivation caused by the acid [7] or to alcohol evaporation [5].

**Figure 3:**
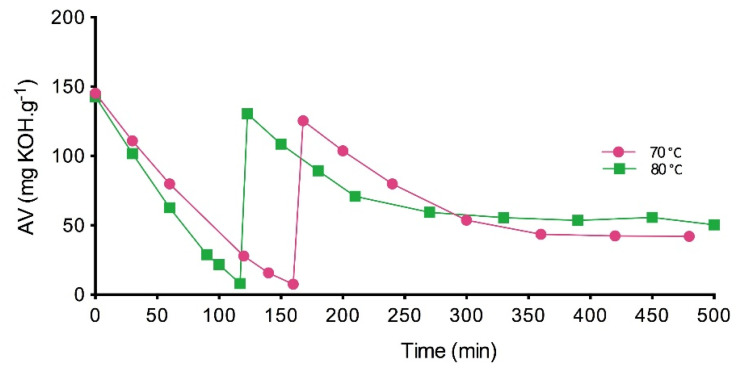
Acid value (AV) with time at 70 °C and 80 °C in a fed‒batch reactor (stepwise acid addition after consumption), 350 rpm, 2.5% (*w/w*) of biocatalyst and a substrate molar ratio of 1:1.

Related to the first hypothesis, several studies have described the irreversible inactivation of CalB caused by the high polarity of short-chain acids, such as heptanoic, butyric, propionic and other acids [7,17,18,19]. It has been demonstrated that lipase is not able to catalyze esterification reactions with acids exhibiting a pKa value less than 4.8. The pKa of MHA is 4.82 ± 0.21 (predicted value) [20]; therefore, it could cause some inactivating effects on the lipase activity. Based on previous studies [7], and in order to increase the final conversion, the acid was added in two equal fractions, the first at the beginning of the reaction and the second when the acid had been consumed. The synthesis was carried out at 70 and 80 °C with a stoichiometric substrate molar ratio and 2.5% (*w/w*) biocatalyst concentration. Acid value (AV) is plotted versus reaction time in Figure 3. After 6 h of reaction, the final acid values were significantly higher than those achieved when the reaction was carried out with a single addition of MHA. In other words, the conversions were substantially lower than those reached with one MHA addition (71 and 64.72% at 70 and 80 °C respectively). These results confirm that the strategy of maintaining a low concentration of acid in the reaction medium does not improve the conversion values previously obtained.

**Figure 4:**
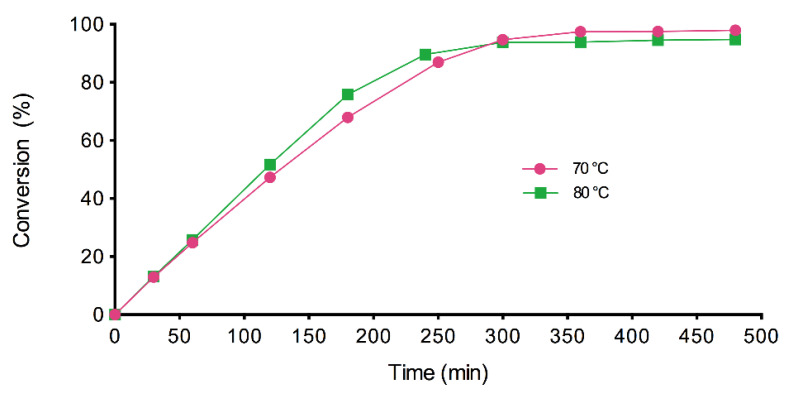
Conversion values of EHMH with time at 70 °C and 80 °C, 350 rpm, 2.5% (*w/w*) of biocatalyst and 10% EH molar excess (1:1.1 substrate molar ratio).

The other explanation for the low conversion values obtained in the preliminary experiments could be the evaporation of the alcohol, which has been observed in previous studies [5]. This hypothesis was confirmed by a GC analysis after 6 h of reaction at 70 and 80 °C, where no EH was identified in the reaction medium and 10% (*w/w*) of MHA was detected. This remaining acid would cause additional problems because the acidity of the cosmetic ingredients is highly restricted and costly separation and purification operations of the final product would be necessary [21]. In previous studies carried out by the authors with EH in other ester syntheses, no alcohol evaporation was detected, probably due to the short reaction time [4]. However, the evaporation of different alcohols was described when the enzymatic reaction was held for 7 h and the loss was compensated for by using a molar excess of alcohol [5,22]. For this reason, a 10% EH molar excess was tried (1:1.1 molar ratio) at both temperatures (70 and 80 °C) and with a single acid addition (Figure 4). As expected, during the first 5 h, the reaction rate was higher at 80 °C than at 70 °C and the conversions, after 6 h, were higher than those obtained with 1:1 molar ratio (97.45 and 93.86% at 70 and 80 °C, respectively). It was also noticed that the final conversion was lower when the temperature was increased, which can be attributed to the larger evaporation of the alcohol at higher temperatures.

**Figure 5:**
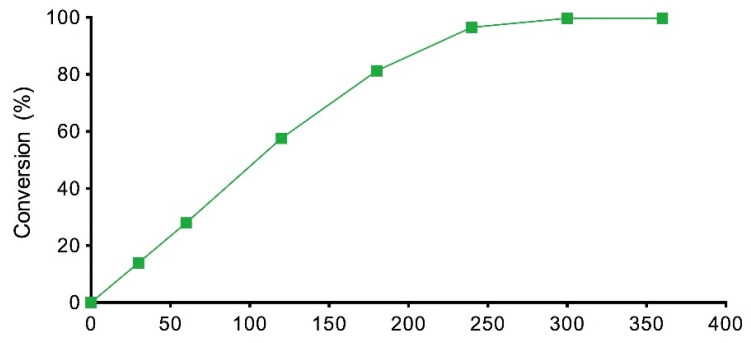
Conversion values of EHMH with time at 80 °C, 350 rpm, 2.5% (*w/w*) of biocatalyst and 20% EH molar excess (1:1.2 substrate molar ratio).

The good results obtained at 70 °C and with a 10% excess of alcohol could be considered as the optimal reaction conditions for the biocatalytic synthesis of EHME. However, the long reaction time that was required (6 h) and the fact that the reaction rate is higher at 80 °C led the authors to try to obtain higher conversions in less time. For this reason, a 20% molar excess of EH was used (1:1.2 molar ratio) at 80 °C. In Figure 5, it can be seen that a conversion of 99.74% is achieved in just 5 h, which is a noticeable improvement, compared to the one obtained previously. However, the use of high temperatures and EH molar excesses could lead to a less environmentally friendly and cost-effective ester production process. Thus, considering a future industrial implementation, the optimal operating conditions selection for the synthesis of EMHE must be based not only on the conversion achieved regarding the reaction time but also on other indicators, such as enzyme reuse, process productivity, green metrics evaluation and economical assessment.

### 3.2. Biocatalyst Reuse and Process Productivity

**Figure 6:**
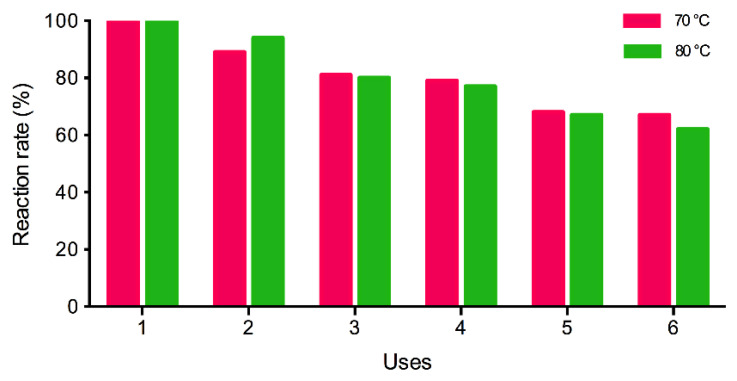
Reusability test of Novozym^®^ 435 for EHMH synthesis at 70 °C, 10% EH excess and 80 °C, 20% EH excess; 350 rpm, 2.5% (*w/w*) biocatalyst.

It is commonly known that enzyme immobilization increases biocatalyst operational stability and makes possible both the operation in continuous reactors and their repetitive use in consecutive batches when they are used in discontinuous processes. This characteristic is essential for a successful industrial implementation of the enzymatic process. The stability of Novozym^®^ 435 after recovery from the reaction medium and its reuse in successive synthesis reactions has been studied under the two operating conditions mentioned above. Figure 6 depicts the results of the reusability assays.

In both experimental series, the immobilized enzyme retains 65–70% of the original activity after six uses. Temperature does not seem to be the main factor that is responsible for this loss, which could be attributed to the low operational stability or biocatalyst manipulation during the recovery step.

EHMH concentrations (% *w/w*) and process productivities have been calculated as a measurement of the real possibilities of an industrial use of the biocatalytic production. Table 2 shows that the higher values of both parameters are obtained when the synthesis is performed at 70 °C and 10% EH excess. Moreover, productivities noticeably increase when the immobilized enzyme is used in six successive reaction cycles and values around 200 are obtained, while EHMH concentrations (% *w/w*) are not affected by the successive uses of lipase. These figures indicate that the process productivity is inside the value ranges described in the literature for the manufacture of fine chemicals or pharma products [23], categories in which EHMH could be included.

### 3.3. Green Metrics and Economical Assessment

In order to help with the selection of the best operating conditions for EHME biocatalytic synthesis and for previously assessing the feasibility of its possible industrial implementation in the context of the principles of “green chemistry” [24], environmental and economic parameters of the two selected processes have also been calculated.

Table 3 lists the most popular and simplest parameters used in green metrics: atom economy (AE), based on the reaction stoichiometry and mechanism [25], E-factor, which analyzes the amount of waste formed in the synthesis of chemical compounds [26] and carbon mass efficiency (CME), which is defined as the percentage of carbon in the reagents that remains in the final product [27].

The AE values are the same for both operating conditions and are very close to 100%, which shows the sustainability of both processes [28]. The other two indicators (E-factor and CME) have been calculated assuming conversions of 97% and 99%, and considering the amount of EH released to the atmosphere. Moreover, the calculated E-factor corresponds to the complete E-factor (cEF), which includes water as waste. The results show that the biocatalytic synthesis of EHMH at 70 °C with 10% EH excess is more sustainable than the other process, due to its higher CME and lower E-factor, the latter being significantly inferior to those described in the literature for the manufacture of fine chemicals, which are usually between 5 and 50 [26].

The preliminary cost estimation of the two processes has been based on the results obtained at laboratory scale. Only direct costs have been considered because they depend on the selected manufacturing method and indirect costs would be common for both processes.

Table 4 shows the prices of the biocatalyst and the substrates (considering their acquisition at bulk scale) provided by their suppliers. Although, in the bibliography, the prices of bulk raw materials are usually estimated by dividing the lab-product price by 10 to 30 [23], we prefer to use some tentative prices given for industrial scale acquisitions. The table also shows the energy cost of the equipment employed, which has been quantified from real-time measurements of the current intensity using a clamp meter. The energy required for the start-up of the system and the maintenance under operating conditions has been calculated assuming that the average voltage of the equipment is 220 V. The price of energy used is 17.5 € kW^−1^×h^−1^ (taxes excluded) [29]. There is an initial consumption of the thermostatic bath to reach the temperature (70 °C or 80 °C) lasting 10 or 14 min, and after that, the temperature control system operates automatically in an auto on/off mode, which implies lower energy consumption.

Once the unit costs for the biocatalyst, the substrates and the process equipment have been obtained, the calculations are carried out to obtain the cost per kilogram of EHMH product [33,34]. Table 5 displays, in the first row, the start-up time to reach the desired temperature and, in the second row, the reaction time until conversion remains constant. The costs per kilogram of product for each item considered are detailed below: biocatalyst, substrates and energy. The results presented in the first two columns correspond to a single use of the immobilized enzyme, whereas the third and fourth columns show the costs when the enzyme is reused six times.

As can be seen in the table, the production of EHMH at 70 °C and 10% EH excess is more profitable than at 80 °C and 20% EH excess, mainly due to the lower energy cost, despite the fact that the operating time is longer. It is worth noting the economic savings produced when the biocatalyst is reused six times, lowering the price per kilogram of EHMH by 63% in the first case and 59% in the second. Under the best operation conditions, the direct operational cost is 21.16 € × kg EHMH^−1^ which is closer to the cost suggested for fine chemicals (15 € × kg^−1^) than for pharmaceutical ingredients (100 € × kg^−1^) [23]. It is important to consider the weight of the biocatalyst cost on the final total product cost, which is recommended to be 10% [23]. In the present study, this percentage increased up to 37%, but could be diminished if the immobilized enzyme were used in more reaction cycles. It can also be observed, in Table 5, that the main impact on the direct cost is due to energy, which supposes more than 50% of the total cost. This can be attributed to the fact that the equipment was connected to a household electrical network and the electricity fee for a standard cosmetic industry supposes almost half of the domestic cost [33].

## 4. Conclusions

This work explores, for the first time, the enzymatic synthesis of 2-ethylhexyl 2-methylhexanoate by using the lipase Novozym^®^ 435 in a solvent-free system. Due to the volatility of the alcohol used as substrate, an excess of 2-ethylhexanol has been added in order to compensate for its evaporation losses and thus achieve higher conversions. Finally, it has been concluded that the synthesis has successfully been developed at 70 °C with a 10% excess of alcohol and at 80 °C with a 20% excess of alcohol, achieving very high conversions in both cases. The final selection was based not only on the conversion reached in the reactor, but also on five additional indexes: enzymatic activity remaining after six uses of the biocatalyst, product concentration, process productivity, green metrics and preliminary direct costs analysis. All the calculated indicators point out the convenience of operating at the lowest temperature and alcohol excess and, under these circumstances, the direct production cost of 2-ethylhexyl 2-methylhexanoate is 21.16 € × kg product^−1^. These results provide the basis for the future industrial implementation of the environmentally friendly process for the production of a new branched-chain acid ester with cosmetic applications.

## Figures and Tables

**Table 1:** Experimental conditions for the assays performed in the batch reactor.

Experiment	Temperature (°C)	Substrates Molar Ratio
Batch reactor	70 and 80	1:1
Fed batch reactor with MHA stepwise addition	70 and 80	1:1
Batch reactor with 10% EH molar excess	70 and 80	1:1.1
Batch reactor with 20% EH molar excess	80	1:1.2

**Table 2:** Product concentrations (% *w/w*) and productivity of the enzymatic synthesis of EHMH at 70 °C, 10% EH molar excess and at 80 °C, 20% EH molar excess, both with a single use and with six uses of the biocatalyst.

Experimental Conditions	Product Concentration (% *w/w*)	Process Productivity (kg EHMH × kg Biocatalyst^−1^)
70 °C, 10% EH excess (biocatalyst single use)	86.40	34.55
80 °C, 20% EH excess (biocatalyst single use)	84.40	33.76
70 °C, 10% EH excess (biocatalyst six uses)	84.93	203.84
80 °C, 20% EH excess (biocatalyst six uses)	82.27	197.46

**Table 3:** Green metrics for the biocatalytic synthesis of EHMH at 70 °C, 10% EH excess and at 80 °C, 20% EH excess; 350 rpm, 2.5% (*w/w*) biocatalyst.

Green Metrics	70 °C, 10% EH Excess	80 °C, 20% EH Excess
Atom economy (AE) * (%)	93.09	93.09
E-factor **	0.16	0.19
Carbon mass efficiency (CME) *** (%)	86.10	83.85

* $AE=\frac{molecular\;weigth\;of\;desired\;product}{\Sigma \;molecular\;weigth\;of\;all\;products}\times 100$, ** $E-factor=\frac{kg\;of\;waste}{kg\;of\;desired\;product}$, *** $CME=\frac{kg\;carbonated\;product}{kg\;carbonated\;reactants}\times 100$.

**Table 4:** Costs of the biocatalyst, substrates and energy.

Materials and Equipment	Cost Item	Prices
Biocatalyst and substrates	Novozym^®^ 435	1600 € × kg^−1^ (Gifted) [30]
	MHA	2.46 € × kg^−1^ [31]
	EH	0.82 € × kg^−1^ [32]
Thermostatic bath (70 °C)	Initial	5.2 × 10^−3^ € × min^−1^
	Maintenance	2 × 10^−4^ € × min^−1^
Thermostatic bath (80 °C)	Initial	5.2 × 10^−3^ € × min^−1^
	Maintenance	6 × 10^−4^ € × min^−1^
Vertical stirrer		10^−4^ € × min^−1^

**Table 5:** Contribution of the biocatalyst, substrates and energy to the direct operation costs of the synthesis of EHMH at laboratory scale with a single use and six uses of enzyme.

Cost Item	70 °C, 10% EH Excess (Biocat. Single Use)	80 °C, 20% EH Excess (Biocat. Single Use)	70 °C, 10% EH Excess (Biocat. Six Uses)	80 °C, 20% EH Excess (Biocat. Six Uses)
Start-up time (min)	10	14	60	84
Reaction time (min)	360	300	2700	2340
Biocatalyst (€ × kg EHMH^−1^)	46.30	47.40	7.85	8.10
Substrates (€ × kg EHMH^−1^)	1.85	1.85	1.88	1.90
Energy (€ × kg EHMH^−1^)	9.59	16.22	11.42	17.03
Total cost (€ × kg EHMH^−1^)	57.75	65.47	21.16	27.03

## Data Availability

The data presented in this study are available upon request from the corresponding author.

## References

[1] Montiel M.C., Máximo F., Serrano-Arnaldos M., Ortega-Requena S., Murcia M.D., Bastida J. (2019). Biocatalytic solutions to cyclomethicones problem in cosmetics. Eng. Life Sci..

[2] Wang L., Zhang Y., Zhang Y., Zheng L., Huang H., Wang Z. (2018). Synthesis of 2-ethylhexyl palmitate catalyzed by enzyme under microwave. Appl. Biochem. Biotechnol..

[3] Haßelberg J., Behr A. (2016). Saturated branched fatty compounds: Proven industrial processes and new alternatives. Eur. J. Lipid Sci. Technol..

[4] Murcia M.D., Serrano-Arnaldos M., Ortega-Requena S., Máximo F., Bastida J., Montiel M.C. (2020). Optimization of a sustainable biocatalytic process for the synthesis of ethylhexyl fatty acids esters. Catal. Today.

[5] Serrano-Arnaldos M., Ortega-Requena S., Sánchez J.A., Hernández A., Montiel M.C., Máximo F., Bastida J. (2021). Sustainable synthesis of branched-chain diesters. J. Biotechnol..

[6] Gómez M., Murcia M.D., Serrano-Arnaldos M., Gómez E., Gómez J.L., Hidalgo A.M., Máximo M.F. (2020). Developing the rate equations for two enzymatic Ping-Pong reactions in series: Application to the bio-synthesis of bis(2-ethylhexyl) azelate. Biochem. Eng. J..

[7] Serrano-Arnaldos M., García-Martínez J.J., Ortega-Requena S., Bastida J., Máximo F., Montiel M.C. (2020). Reaction strategies for the enzymatic synthesis of neopentyl glycol diheptanoate. Enzyme Microb. Technol..

[8] Su G.D., Huang D.F., Han S.Y., Zheng S.P., Lin Y. (2010). Display of *Candida antarctica* lipase B on *Pichia pastoris* and its application to flavor ester synthesis. Appl. Microbiol. Biotechnol..

[9] Keng P.S., Basri M., Zakaria M.R.S., Rahman M.B.A., Ariff A.B., Rahman R.N.Z.A., Salleh A.B. (2009). Newly synthesized palm esters for cosmetics industry. Ind. Crops Prod..

[10] Daneshfar A., Ghaziaskar H.S., Shiri L., Manafi M.H., Nikorazm M., Abassi S. (2007). Synthesis of 2-ethylhexyl-2-ethylhexanoate catalyzed by immobilized lipase in n-hexane: A kinetic study. Biochem. Eng. J..

[11] Chen H.C., Kuo C.-H., Chen H.H., Liu Y.-C., Shieh C.J. (2011). Optimization of enzymatic synthesis of cetyl 2-ethylhexanoate by Novozym^®^ 435. J. Am. Oil Chem. Soc..

[12] Ghaziaskar H.S., Daneshfar A., Calvo L. (2006). Continuous esterification or dehydration in supercritical carbon dioxide. Green Chem..

[13] Juhl P.B., Doderer K., Hollmann F., Thum O., Pleiss J. (2010). Engineering of *Candida antarctica* lipase B for hydrolysis of bulky carboxylic acid esters. J. Biotechnol..

[14] ASTM D974-02e1 (2002). Standard Test Method for Acid and Base Number by Color-Indicator Titration.

[15] Ortiz C., Ferreira M.L., Barbosa O., dos Santos J.C.S., Rodrigues R.C., Berenguer-Murcia Á., Briand L.E., Fernandez-Lafuente R. (2019). Novozym 435: The “perfect” lipase immobilized biocatalyst?. Catal. Sci. Technol..

[16] Global Insight (2007). A Study of the European Cosmetics Industry. Final Report.

[17] de Meneses A.C., Almeida Sá A.G., Lerin L.A., Corazza M.L., de Araújo P.H.H., Sayer C., de Oliveira D. (2019). Benzyl butyrate esterification mediated by immobilized lipases: Evaluation of batch and fed-batch reactors to overcome lipase-acid deactivation. Process Biochem..

[18] Hollmann F., Grzebyk P., Heinrichs V., Doderer K., Thum O. (2009). On the inactivity of *Candida antartica* lipase B towards strong acids. J. Mol. Catal. B Enzym..

[19] Nordblad M., Adlercreutz P. (2008). Effects of acid concentration and solvent choice on enzymatic acrylation by *Candida antarctica* lipase B. J. Biotechnol..

[20] 2-Methylhexanoic Acid Properties. https://www.chemicalbook.com/ChemicalProductProperty_EN_CB1421374.htm.

[21] Heath R.S., Ruscoe R.E., Turner N.J. (2021). The beauty of biocatalysis: Sustainable synthesis of ingredients in cosmetics. Nat. Prod. Rep..

[22] Lee A., Kim H., Choi N., Yoon S.W., Kim Y., Kim H.-R., Kim I.-H. (2019). Preparation of diisononyl adipate in a solvent-free system via an immobilized lipase-catalyzed esterification. Enzyme Microb. Technol..

[23] Tufvesson P., Lima-Ramos J., Nordblad M., Woodley J. (2011). Guidelines and cost analysis for catalyst production in biocatalytic processes. Org. Process Res. Dev..

[24] Anastas P., Eghbali N. (2010). Green chemistry: Principles and practice. Chem. Soc. Rev..

[25] Sheldon R. (2000). Atom efficiency and catalysis in organic synthesis. Pure Appl. Chem..

[26] Sheldon R.A. (2017). The E factor 25 years on: The rise of green chemistry and sustainability. Green Chem..

[27] Lima Ramos J., Tufvesson P., Woodley J. (2014). Application of environmental and economic metrics to guide the development of biocatalytic processes. Green Process. Synth..

[28] Augé J. (2008). A new rationale of reaction metrics for green chemistry. Mathematical expression of the environmental impact factor of chemical processes. Green Chem..

[29] Comisión Nacional de los Mercados y la Competencia (2021). Boletín de Indicadores eléctricos de Julio de 2021. https://www.cnmc.es/sites/default/files/3644997_0.pdf.

[30] https://www.novozymes.com/en/advance-your-business/pharma.

[31] https://m.lookchem.com/casno4536-23-6.html.

[32] https://spanish.alibaba.com/product-detail/industrial-2-ethylhexanol-2eh-2-ethyl-hexanol-for-sale-1600225851350.html?spm=a2700.shop_plser.41413.24.517c5592ca093M.

[33] Serrano-Arnaldos M., Montiel M.C., Ortega-Requena S., Máximo F., Bastida J. (2020). Development and economic evaluation of an eco-friendly biocatalytic synthesis of emollient esters. Bioproc. Biosyst. Eng..

[34] Serrano-Arnaldos M., Ortega-Requena S., Montiel M.C., Máximo F., Bastida J., Murcia M.D. (2019). Preliminary economic assessment: A valuable tool to establish biocatalytic process feasibility with an in-lab immobilized lipase. J. Chem. Technol. Biotechnol..

